# Antiviral drugs arbidol and interferon alpha-1b contribute to reducing the severity of COVID-19 patients: a retrospective cohort study

**DOI:** 10.1186/s12985-021-01617-w

**Published:** 2021-07-08

**Authors:** Peng Yin, Juan Meng, Jincheng Chen, Junxiao Gao, Dongqi Wang, Shuyan Liu, Qinglong Guo, Muchun Zhu, Gengwei Zhang, Yingxia Liu, Ye Li, Guoliang Zhang

**Affiliations:** 1 Shenzhen Institute of Advanced Technology, Chinese Academy of Sciences, Shenzhen, 518055 China; 2 National Clinical Research Center for Infectious Diseases, Shenzhen Third People’s Hospital, Southern University of Science and Technology, Shenzhen, 518112 China; 3 Savaid Medical School, University of Chinese Academy of Sciences, Beijing, 100049 China

**Keywords:** Arbidol, Interferon alpha-1b, Severity rate, COVID-19, Antiviral agents

## Abstract

**Objectives:**

The aim of this study was to evaluate the role of antiviral drugs in reducing the risk of developing severe illness in patients with moderate COVID-19 pneumonia.

**Methods:**

This retrospective cohort study included 403 adult patients with moderate COVID-19 pneumonia who were admitted to Shenzhen Third People’s Hospital, China. The antiviral drugs arbidol, interferon alpha-1b, lopinavir–ritonavir and ribavirin were distributed to the patients for treatment. The primary endpoint of this study was the time to develop severe illness.

**Results:**

Of the 462 patients admitted, 403 had moderate COVID-19 symptoms at hospital admission and were included in this study. 90 of the 403 (22.3%) patients progressed to severe illness. The use of arbidol was associated with a lower severity rate 3.5% compared to control group 30.5%, *p*-value < 0.0001; the adjusted hazard ratio was 0.28 (95% CI: 0.084–0.90, *p* = 0.033). The use of interferon alpha-1b was associated with a lower severity rate 15.5% compared to control group 29.3%, with *p*-value < 0.0001; the adjusted hazard ratio was 0.30 (95% CI: 0.15–0.58, *p* =  0.0005). The use of lopinavir–itonavir and ribavirin did not show significant differences in adjusted regression models. Early use of arbidol within 7 days of symptom onset was significantly associated with a reduced recovery time of − 5.2 days (IQR − 3.0 to − 7.5, *p* = 4e−06) compared with the control group.

**Conclusion:**

Treatment with arbidol and interferon alpha-1b contributes to reducing the severity of illness in patients with moderate COVID-19 pneumonia. Early use of arbidol may reduce patients’ recovery time.

**Supplementary Information:**

The online version contains supplementary material available at 10.1186/s12985-021-01617-w.

## Background

Severe acute respiratory syndrome coronavirus 2 (SARS-CoV-2) has been detected since December 2019 and confirmed of person-to-person transmission. The coronavirus disease 2019 (COVID-19) pandemic has affected more than 127 million patients with more than 2.7 million deaths in more than 230 countries and regions [1]. Drug repurposing has been proposed as an option to fight SARS-CoV-2, for example, the antiviral drugs glycyrrhizin and ribavirin used to combat SARS and MERS, the family of coronaviruses [2, 3]. A number of clinical trials have been performed since the outbreak of SAS-COV-2. Several popular drugs have been tested, including chloroquine/hydroxychloroquine, lopinavir and ritonavir, arbidol, ribavirin, and azithromycin, etc. Sanders et al. and pharmacists in Elsevier's Clinical Solutions group reviewed current evidence regarding major proposed treatments, repurposed or experimental information for COVID-19 and provided a summary of contemporary clinical experience and treatment guidance for this novel epidemic coronavirus [4, 5]. They investigated multiple drugs that were already in clinical use, for instance, remdesivir, chloroquine, favipiravir, lopinavir, and anticoagulation, and listed a variety of clinical drug details. However, the efficiency of using approved antiviral drugs to treat COVID-19 patients remains unclear (Table 1).

**Table 1:** Literature review of COVID-19 clinical trials with antiviral drugs

Antiviral drug	Literature	Findings	Study type
Hydroxychloroquine with or without azithromycin	Cavalcanti et al. [8]	Did not improve clinical status at 15 days for mild-to-moderate COVID-19	Open-label RCT
Remdesivir	Beigel et al. [11]	Shorten the time to recovery for hospitalized patients	Double-blind RCT
	Wang et al. [14]	Was not associated with time to clinical improvement	Double-blind RCT
Ribavirin	Tong et al. [15]	Was not associated with improved negative conversion time/improved mortality rate for severe patients	Retrospective study
Lopinavir–ritonavir (LPV/r)	Cao et al. [16]	Did not significantly accelerate clinical improvement for severe patients	Open-label RCT
Interferon beta-1b + lopinavir–ritonavir + ribavirin	Hung et al. [17]	Alleviating symptoms and shortening the duration of viral shedding and hospital stay in patients with mild to moderate COVID-19	Open-label RCT
Arbidol vs. lopinavir–ritonavir	Zhu et al. [18]	Arbidol monotherapy may be superior to lopinavir/ritonavir for viral clearance	Retrospective study
Arbidol + lopinavir–ritonavir vs. lopinavir–ritonavir	Deng et al. [19]	Arbidol and LPV/r combination may be superior to LPV/r alone in elevating negative conversion rate	Retrospective study
Arbidol and moxifloxacin	Yu et al. [20]	Reducing viral load and inflammation	Retrospective study
Empirical antiviral regimens with or without arbidol	Xu et al. [21]	Arbidol could accelerate and enhance the process of viral clearance	Retrospective study
Tocilizumab	Eimer et al. [24]	Did not reduce all-cause mortality but was associated with a shorter time on mechanical ventilation	Retrospective study
Dexamethasone	RECOVERY Collaborative group [25]	Lower 28-day mortality among those who were receiving either invasive mechanical ventilation or oxygen alone but not among those receiving no respiratory support	Open-label RCT

We list the main findings of previous works

*RCT* randomized controlled trial

A number of clinical trials using chloroquine/hydroxychloroquine have been conducted [6–8]. For example, Cavalcanti et al. conducted a multicenter, randomized, open-label, three-group, controlled trial and discovered that the use of hydroxychloroquine, alone or with azithromycin, did not improve clinical status at 15 days compared with standard care among patients hospitalized with mild-to-moderate COVID-19 [8]. Other evidence in animal models has been found as well [9, 10].

A more promising drug, remdesivir, which was designed to treat Ebola, was tested and given emergency use authorization by the FDA, although contradictions still exist [11–14]. For example, Beigel et al. showed that remdesivir was superior to the placebo in shortening the time to recovery in adults hospitalized with COVID-19, and in providing evidence of lower respiratory tract infection [11]. Rochwerg et al. found that remdesivir may be effective in reducing the time to clinical improvement and may decrease mortality in patients with severe COVID-19 [12]. On the other hand, Wang et al. revealed that remdesivir was not associated with statistically significant clinical benefits in a randomized, double-blind, placebo-controlled, multicenter trial [14].

Ribavirin, lopinavir–ritonavir and arbidol were also pinpointed and tested in a number of clinical trials. Tong et al. discovered that ribavirin therapy was not associated with improved negative conversion time for the SARS-CoV-2 test and was not associated with an improved mortality rate in patients with severe COVID-19 [15]. Cao et al. found that lopinavir–ritonavir treatment did not significantly accelerate clinical improvement, reduce mortality, or diminish throat viral RNA detectability in patients with serious COVID-19 [16].

The combination of multiple antiviral therapies has also been tested. For example, Hung et al. demonstrated that early treatment with the triple combination of antiviral therapy with interferon beta-1b, lopinavir–ritonavir, and ribavirin was safe and highly effective in shortening the duration of virus shedding, decreasing cytokine responses, alleviating symptoms, and facilitating the discharge of patients with mild to moderate COVID-19 [17]. The triple antiviral therapy rapidly rendered the viral load negative in all specimens, thereby reducing the patients’ infectiousness. Zhu et al. evaluated the antiviral effects and safety of lopinavir/ritonavir and arbidol in patients with 2019-nCoV disease, and suggested that arbidol monotherapy may be superior to lopinavir/ritonavir in treating COVID-19 [18]. Deng et al. found that oral arbidol and lopinavir/ritonavir in the combination group were associated with a significantly elevated negative conversion rate of the coronavirus test at 7 days and 14 days, compared with lopinavir/ritonavir only in the monotherapy group [19]. Yu et al. argued that the treatment of arbidol and moxifloxacin could be helpful in reducing viral load and inflammation during SARS-CoV-2 infection, especially for negatively regulating fatal inflammation in severe COVID-19 patients [20]. Xu et al. performed a retrospective cohort study of COVID-19 patients who received empirical antiviral regimens with or without arbidol, and implied that arbidol could accelerate and enhance the process of viral clearance, improve focal absorption in radiologic images, and alleviate the demand for oxygen therapy in hospitalization [21]. Apart from clinical trials, Wang et al. assessed six currently available and licensed anti-influenza drugs (arbidol, baloxavir, laninamivir, oseltamivir, peramivir, and zanamivir) against SARS-CoV-2, and discovered that arbidol is an efficient inhibitor of SARS-CoV-2 in vitro and appears to block virus entry by impeding viral attachment and release from ELs [22].

To treat severe patients, Zhang et al. summarized the current evidence and shared their experience in anti-inflammatory treatment, including glucocorticoids, IL-6 antagonists, JAK inhibitors and chloroquine/hydrocholoroquine, of patients with severe COVID-19 who may have an impaired immune system [23]. Eimer et al. indicated that treatment with tocilizumab in critically ill patients with severe ARDS due to COVID-19 may lessen the time on mechanical ventilation and the overall length of stay in the ICU and in the hospital [24]. The RECOVERY Collaborative group found that the use of dexamethasone resulted in lower 28-day mortality among those who were receiving either invasive mechanical ventilation or oxygen alone at randomization but not among those receiving no respiratory support [25].

Antiviral treatments including interferon alpha, lopinavir–ritonavir, chloroquine phosphate, ribavirin, and arbidol, have been included in the latest version of the Guidelines for the Prevention, Diagnosis, and Treatment of Novel Coronavirus-induced Pneumonia issued by the National Health Commission (NHC) of the People’s Republic of China for the tentative treatment of COVID-19 [26]. We reviewed the cohort of 462 confirmed COVID-19 cases in Shenzhen China, who were admitted to The Third People’s Hospital of Shenzhen. Antiviral drugs including arbidol, interferon alpha-1b, lopinavir–ritonavir and ribavirin were used to treat COVID-19 patients.

## Methods

### Study design and patients

This is a retrospective cohort study. Between 11 January 2020 and 21 May 2020, total 462 confirmed cases were admitted to The Third People’s Hospital of Shenzhen. One patient was in critical illness at hospital admission; two patients were in severe illness and further progressed to respiratory failure. The other 30 cases including eight children were in a mild condition, with no severe disease progression. A total of 429 cases had moderate illness, including 26 children.

**Fig. 1:**
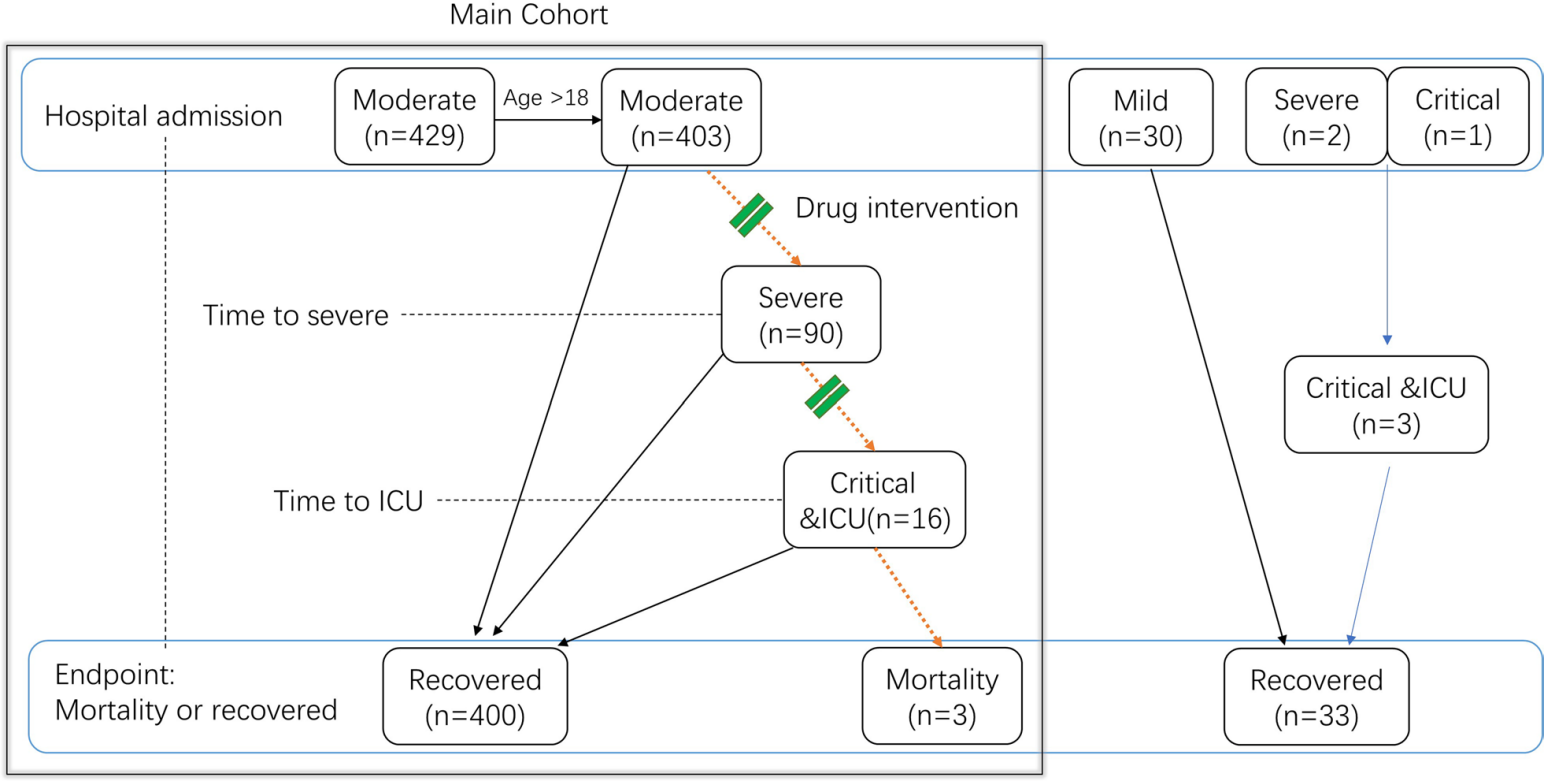
Design of the cohort analysis. Of total 462 cases, 403 adult patients in moderate illness at hospital admission were included in the main cohort study

We included 403 moderate adult patients at hospital admission as the main cohort of the study (Fig. 1). Mild cases were not included as none developed severe illness. All patients were followed up to the endpoint: mortality or recovery and discharge from hospital.

### Procedures

The majority of patients were given antiviral drugs as common interventions. In January 2020, when patients started to enroll into hospital, ribavirin and interferon alpha-1b were distributed to patients according to experience from SARS [26]. In late January 2020, patients started to use lopinavir–ritonavir; in early February 2020, patients started to use arbidol.

Drugs were given in regular doses. Ribavirin was given by injection at dose of 300–500 mg twice per day or by oral pills at daily dose of 500–900 mg for at least three days. Interferon alpha-1b was used at dose of 50–60 µg twice per day by injection for at least three days. Oral lopinavir–ritonavir was used at dose of 500 mg (lopinavir 400 mg and ritonavir 100 mg) twice per day for at least three days. Oral arbidol was used at dose of 200 mg three times per day for at least three days.

Other interventions included oxygen support for severe patients using non-invasive and invasive ventilatory support, and antimicrobial treatment for bacterial infection as indicated clinically.

### Statistical analysis

For each drug, we separated the patients into two groups: taking the drug (treatment) or not taking the drug (control). We used a logistic regression test to compare the two groups of data.

The Kaplan–Meier (KM) test was used for single factor comparations, including age (≥ median age 48; < 48), sex (male vs female), and drug use. The *p*-value was calculated by the KM test, and standard survival curves of the cumulative hazard of developing severity were plotted.

The multivariable associations between primary outcome (time to events) and drug use and other variables were further tested by the Cox proportional hazard model. Variables with significant associations were included and adjusted with *p*-value < 0.1. Variables related to drug use were also included to avoid confounding bias. Similar analysis was performed for secondary outcome. Sensitivity analysis was performed and the results were robust.

For hospitalized length evaluation, we used the fixed effect linear regression model. Risk factors were adjusted with *p*-value < 0.1. All statistical analyses were performed using R software version 4.0.1.

## Results

### Descriptive demographics

The patients’ demographic information included age, sex, disease history and clinical symptoms (Table 2). The patients’ median age was 48 years with inter-quantile range (35, 60). About half were male (203), and the other half were female (200). Fever (70%), cough (53%) and fatigue (19%) were common symptoms at onset. Many patients had a disease history, mainly hypertension (61), diabetes (19), cardiovascular disease history (19), respiratory disease history (21) and other infectious disease histories (17).

**Table 2:** Descriptive demographics of COVID-19 patients across treatment groups

		Lopinavir–ritonavir			IFN-α			Arbidol			Ribavirin		
	Total (n = 403)	Yes (n = 335)	No (n = 68)	*p*-value	Yes (n = 341)	No (n = 62)	*p*-value	Yes (n = 151)	No (n = 252)	*p*-value	Yes (n = 112)	No (n = 291)	*p*-value
Age (yrs, median, IQR)	48[35, 60]	49[35, 61]	45[35, 57]	0.42	49[35, 61]	43[34, 56]	0.071	46[33, 61]	49[36, 60]	0.23	56[40, 64]	46[32, 57]	6.1E−07
Sex	M:203 (50.4%) F:200 (49.6%)	M:169 (50.4%) F:166 (49.6%)	M:34 (50.0%) F:34 (50.0%)	0.95	M:169 (49.6%) F:172 (50.4%)	M:34 (54.8%) F:28 (45.2%)	0.44	M:84 (55.6%) F:67 (44.4%)	M:119 (47.2%) F:133 (52.8%)	0.10	M:65 (58.0%) F:47 (42.0%)	M:138 (47.4%) F:153 (52.6%)	0.056
Fever #No. of cases (%)	Y:282 (70.0%) N:121 (30.0%)	Y:247 (73.7%) N:88 (26.3%)	Y:35 (51.5%) N:33 (48.5%)	2.4E−04	Y:254 (74.5%) N:87 (25.5%)	Y:28 (45.2%) N:34 (54.8%)	2.8E−06	Y:100 (66.2%) N:51 (33.8%)	Y:182 (72.2%) N:70 (27.8%)	0.21	Y:85 (75.9%) N:27 (24.1%)	Y:197 (67.7%) N:94 (32.3%)	0.11
Cough	Y:214 (53.1%) N:189 (46.9%)	Y:182 (54.3%) N:153 (45.7%)	Y:32 (47.1%) N:36 (52.9%)	0.27	Y:179 (52.5%) N:162 (47.5%)	Y:35 (56.5%) N:27 (43.5%)	0.57	Y:73 (48.3%) N:78 (51.7%)	Y:141 (56.0%) N:111 (44.0%)	0.14	Y:72 (64.3%) N:40 (35.7%)	Y:142 (48.8%) N:149 (51.2%)	0.005
Fatigue	Y:76 (18.9%) N:327 (81.1%)	Y:61 (18.2%) N:274 (81.8%)	Y:15 (22.1%) N:53 (77.9%)	0.46	Y:70 (20.5%) N:271 (79.5%)	Y:6 (9.7%) N:56 (90.3%)	0.045	Y:20 (13.2%) N:131 (86.8%)	Y:56 (22.2%) N:196 (77.8%)	0.026	Y:27 (24.1%) N:85 (75.9%)	Y:49 (16.8%) N:242 (83.2%)	0.095
Running nose	Y:44 (10.9%) N:359 (89.1%)	Y:32 (9.6%) N:303 (90.4%)	Y:12 (17.6%) N:56 (82.4%)	0.051	Y:37 (10.9%) N:304 (89.1%)	Y:7 (11.3%) N:55 (88.7%)	0.92	Y:20 (13.2%) N:131 (86.8%)	Y:24 (9.5%) N:228 (90.5%)	0.25	Y:16 (14.3%) N:96 (85.7%)	Y:28 (9.6%) N:263 (90.4%)	0.18
Headache	Y:68 (16.9%) N:335 (83.1%)	Y:54 (16.1%) N:281 (83.9%)	Y:14 (20.6%) N:54 (79.4%)	0.37	Y:56 (16.4%) N:285 (83.6%)	Y:12 (19.4%) N:50 (80.6%)	0.57	Y:27 (17.9%) N:124 (82.1%)	Y:41 (16.3%) N:211 (83.7%)	0.68	Y:19 (17.0%) N:93 (83.0%)	Y:49 (16.8%) N:242 (83.2%)	0.97
Diarrhea	Y:39 (9.7%) N:364 (90.3%)	Y:35 (10.4%) N:300 (89.6%)	Y:4 (5.9%) N:64 (94.1%)	0.25	Y:33 (9.7%) N:308 (90.3%)	Y:6 (9.7%) N:56 (90.3%)	1.00	Y:14 (9.3%) N:137 (90.7%)	Y:25 (9.9%) N:227 (90.1%)	0.83	Y:13 (11.6%) N:99 (88.4%)	Y:26 (8.9%) N:265 (91.1%)	0.42
Nausea	Y:13 (3.2%) N:390 (96.8%)	Y:11 (3.3%) N:324 (96.7%)	Y:2 (2.9%) N:66 (97.1%)	0.88	Y:11 (3.2%) N:330 (96.8%)	Y:2 (3.2%) N:60 (96.8%)	1.00	Y:4 (2.6%) N:147 (97.4%)	Y:9 (3.6%) N:243 (96.4%)	0.61	Y:6 (5.4%) N:106 (94.6%)	Y:7 (2.4%) N:284 (97.6%)	0.13
Respiratory symptom	Y:18 (4.5%) N:385 (95.5%)	Y:16 (4.8%) N:319 (95.2%)	Y:2 (2.9%) N:66 (97.1%)	0.51	Y:15 (4.4%) N:326 (95.6%)	Y:3 (4.8%) N:59 (95.2%)	0.88	Y:6 (4.0%) N:145 (96.0%)	Y:12 (4.8%) N:240 (95.2%)	0.71	Y:7 (6.2%) N:105 (93.8%)	Y:11 (3.8%) N:280 (96.2%)	0.28
Pharyngalgia	Y:71 (17.6%) N:332 (82.4%)	Y:62 (18.5%) N:273 (81.5%)	Y:9 (13.2%) N:59 (86.8%)	0.30	Y:61 (17.9%) N:280 (82.1%)	Y:10 (16.1%) N:52 (83.9%)	0.74	Y:29 (19.2%) N:122 (80.8%)	Y:42 (16.7%) N:210 (83.3%)	0.52	Y:18 (16.1%) N:94 (83.9%)	Y:53 (18.2%) N:238 (81.8%)	0.61
Myalgia	Y:54 (13.4%) N:349 (86.6%)	Y:51 (15.2%) N:284 (84.8%)	Y:3 (4.4%) N:65 (95.6%)	0.017	Y:49 (14.4%) N:292 (85.6%)	Y:5 (8.1%) N:57 (91.9%)	0.18	Y:22 (14.6%) N:129 (85.4%)	Y:32 (12.7%) N:220 (87.3%)	0.60	Y:22 (19.6%) N:90 (80.4%)	Y:32 (11.0%) N:259 (89.0%)	0.022
Chest symptom	Y:39 (9.7%) N:364 (90.3%)	Y:33 (9.9%) N:302 (90.1%)	Y:6 (8.8%) N:62 (91.2%)	0.79	Y:33 (9.7%) N:308 (90.3%)	Y:6 (9.7%) N:56 (90.3%)	1.00	Y:11 (7.3%) N:140 (92.7%)	Y:28 (11.1%) N:224 (88.9%)	0.21	Y:16 (14.3%) N:96 (85.7%)	Y:23 (7.9%) N:268 (92.1%)	0.052
Hypertension history	Y:61 (15.1%) N:342 (84.9%)	Y:52 (15.5%) N:283 (84.5%)	Y:9 (13.2%) N:59 (86.8%)	0.63	Y:51 (15.0%) N:290 (85.0%)	Y:10 (16.1%) N:52 (83.9%)	0.81	Y:25 (16.6%) N:126 (83.4%)	Y:36 (14.3%) N:216 (85.7%)	0.54	Y:21 (18.8%) N:91 (81.2%)	Y:40 (13.7%) N:251 (86.3%)	0.21
Cardiovascular history	Y:19 (4.7%) N:384 (95.3%)	Y:16 (4.8%) N:319 (95.2%)	Y:3 (4.4%) N:65 (95.6%)	0.89	Y:16 (4.7%) N:325 (95.3%)	Y:3 (4.8%) N:59 (95.2%)	0.96	Y:8 (5.3%) N:143 (94.7%)	Y:11 (4.4%) N:241 (95.6%)	0.67	Y:7 (6.2%) N:105 (93.8%)	Y:12 (4.1%) N:279 (95.9%)	0.37
Respiratory History	Y:21 (5.2%) N:382 (94.8%)	Y:20 (6.0%) N:315 (94.0%)	Y:1 (1.5%) N:67 (98.5%)	0.13	Y:16 (4.7%) N:325 (95.3%)	Y:5 (8.1%) N:57 (91.9%)	0.27	Y:7 (4.6%) N:144 (95.4%)	Y:14 (5.6%) N:238 (94.4%)	0.69	Y:12 (10.7%) N:100 (89.3%)	Y:9 (3.1%) N:282 (96.9%)	0.002
Diabetes history	Y:19 (4.7%) N:384 (95.3%)	Y:15 (4.5%) N:320 (95.5%)	Y:4 (5.9%) N:64 (94.1%)	0.62	Y:17 (5.0%) N:324 (95.0%)	Y:2 (3.2%) N:60 (96.8%)	0.55	Y:4 (2.6%) N:147 (97.4%)	Y:15 (6.0%) N:237 (94.0%)	0.13	Y:6 (5.4%) N:106 (94.6%)	Y:13 (4.5%) N:278 (95.5%)	0.71
Infection disease history	Y:17 (4.2%) N:386 (95.8%)	Y:16 (4.8%) N:319 (95.2%)	Y:1 (1.5%) N:67 (98.5%)	0.22	Y:14 (4.1%) N:327 (95.9%)	Y:3 (4.8%) N:59 (95.2%)	0.79	Y:5 (3.3%) N:146 (96.7%)	Y:12 (4.8%) N:240 (95.2%)	0.48	Y:5 (4.5%) N:107 (95.5%)	Y:12 (4.1%) N:279 (95.9%)	0.88
Allergic history	Y:38 (9.4%) N:365 (90.6%)	Y:33 (9.9%) N:302 (90.1%)	Y:5(7.4%) N:63 ( (92.6%)	0.52	Y:34 (10.0%) N:307 (90.0%)	Y:4 (6.5%) N:58 (93.5%)	0.38	Y:18 (11.9%) N:133 (88.1%)	Y:20 (7.9%) N:232 (92.1%)	0.19	Y:10 (8.9%) N:102 (91.1%)	Y:28 (9.6%) N:263 (90.4%)	0.83
Surgery history	Y:76 (18.9%) N:327 (81.1%)	Y:68 (20.3%) N:267 (79.7%)	Y:8 (11.8%) N:60 (88.2%)	0.10	Y:60 (17.6%) N:281 (82.4%)	Y:16 (25.8%) N:46 (74.2%)	0.13	Y:33 (21.9%) N:118 (78.1%)	Y:43 (17.1%) N:209 (82.9%)	0.24	Y:18 (16.1%) N:94 (83.9%)	Y:58 (19.9%) N:233 (80.1%)	0.38

Drug use was evaluated by comparing groups of patients taking (Yes) or not taking the drug (No) during their hospital stay. The patients’ demographic information included age, sex, disease history and clinical symptoms

Drug use was evaluated by comparing groups of patients taking (Yes) or not taking the drug (No) during their hospital stay. As shown in Table 2, there was no apparent difference between the drug-use group and the control group for most cases. To spot some lack of randomization, ribavirin was given more frequently for older patients than younger patients. The fever group had more patients taking interferon alpha-1b (IFN-α) and lopinavir–ritonavir. The fatigue group had more patients taking IFN-α but few patients taking arbidol. The cough and respiratory disease history groups had more patients taking ribavirin. To avoid confounding bias, we adjusted these factors in the multivariable models.

### Outcomes

First, we used the 403 patients’ data with time to developing severity as the main clinical outcome, where the start timepoint was the date of symptoms onset and the endpoint was the date of developing severity if patients developed severe illness (90 patients) or the date of hospital discharge if patients did not develop severe illness before recovery (313 patients).

Second, among the 90 patients who developed severe disease, 16 further progressed worse to respiratory failure and required mechanical ventilation and were admitted to the ICU. The secondary outcome was the time to develop critical illness from severe illness.

Third, apart from three mortality cases, the remaining patients recovered. We also evaluated the patients’ recovery time, defined as the time length (in days) between symptoms onset and hospital discharge, and in-hospital time, defined as the time length between hospital admission and hospital discharge.

### Primary outcome

**Fig. 2:**
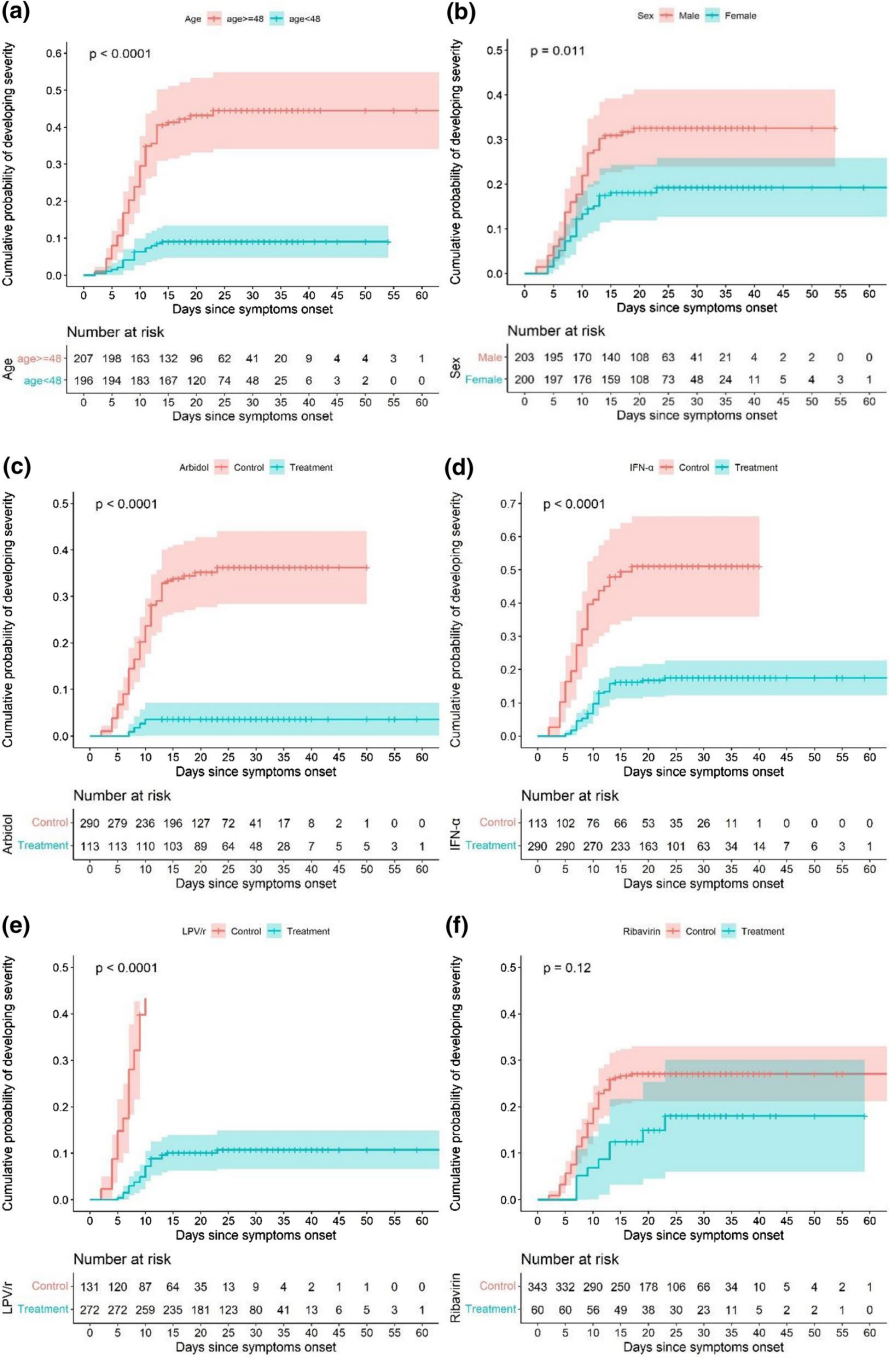
Survival curves for the primary outcome (time to severity). Comparative groups between **a** age < 48 years vs age ≥ 48, **b** male vs female, **c** arbidol vs control, **d** interferon alpha-1b (IFN-α) vs control, **e** lopinavir–ritonavir (LPV/r) vs control, **f** ribavirin vs control

We first tested the association between primary outcome and age, sex, and drug use with the Kaplan–Meier test. The cumulative hazard of developing severity was plotted for age, sex and drug use. Significant differences between the groups were discovered with *p*-value < 0.05. As seen in Fig. 2a, b, older age and male population were more risky than younger and female population. As shown in Fig. 2c–f, the use of antiviral drugs seems to benefit the patients.

Next, we tested the joint association between the primary outcome and drug use by the Cox proportional hazard model, adjusting for risk factors. The results are summarized in Table 3. Older age was significantly associated with increased risk, hazard ratio: 1.046 (95% CI:1.026–1.066, *p*-value: 3.1e−06). Females were at lower risk than males. Symptoms onset and cardiovascular disease history may indicate high risk. For drug intervention, the use of arbidol and IFN-α was significantly associated with a reduced risk of developing severity. Arbidol has lowered the risk by 72% (95% CI: 10 to 92%; *p*-value: 0.033). IFN-α has lowered the risk by 70% (95% CI: 42% to 85%; *p*-value: 0.0005). Ribavirin and lopinavir–ritonavir were not significantly associated with reduced risk, although the trend was still positive.

**Table 3:** Primary outcome (time to severity) association with drug use

	Hazard ratio (95% CI)	*P*-value
Age (years)	1.046 (1.026, 1.066)	3.1E-06
Sex (Female)	0.71 (0.43, 1.15)	0.17
Fever	2.77 (1.31, 5.85)	0.007
Cough	1.32 (0.81, 2.18)	0.26
Fatigue	1.24 (0.77, 2.00)	0.37
Respiratory symptom	1.34 (0.59, 3.05)	0.48
Cardiovascular disease history	3.91 (1.82, 8.38)	0.0004
Arbidol*	0.28 (0.084, 0.90)	0.033
Ribavirin	0.53 (0.24, 1.16)	0.11
IFN-α*	0.30 (0.15, 0.58)	0.0005
Lopinavir–ritonavir	0.54 (0.28, 1.01)	0.055

Survival analysis was performed to test the association between the primary outcome and drug use by the Cox proportional hazard model. Risk factors includes age, sex, clinical symptoms, disease history were adjusted

Sensitivity analysis were performed to test drug-drug interaction in the regression model and the results are shown in Additional file 1: Table S4. No significant drug-drug interactions were detected. We also included the stratified cohort that with only one kind of drugs to compare with those without any drugs. Arbidol, IFN-α and lopinavir–ritonavir were significantly associated with the primary outcome, despite of the small sample sizes. The effect of IFN-α was further confirmed by multivariate regression model (Additional file 1: Table S5).

### Secondary outcome

For the 90 patients who developed severe disease, 16 further progressed to respiratory failure and were admitted to the ICU on average of 2.5 (IQR: 0 to 3) days. Drug intervention for severe patients showed no significant efficacy (as shown in Additional file 1: Table S1). Apart from older age, respiratory symptom onset was significantly associated with an increased risk of developing critical illness, which may indicate that these patients should be monitored carefully.

### Time length in hospital

Apart from the three mortality cases, the remaining patients recovered and were discharged from hospital on average of 25 days (IQR: 19 to 34 days). A multivariate linear regression model was used to test the association between time length and drug use; with several risk factors adjusted: age, sex, symptoms onset, disease history, epidemiology factor and changes in hospitalized guidelines.

The results are shown in Table 4. There were no significantly differences in the use of arbidol or IFN-α compared with controls, while the use of ribavirin and lopinavir–ritonavir delayed patient recovery. As stated by a previous study, early intervention may be crucial (also see Additional file 1: Table S3) [15]. We further separated the treatment group into an early use group (taking drugs within 7 days of symptoms onset) and a late use group (taking drugs after 7 days of symptoms onset). We examined the association by a linear regression model. The early use of arbidol was significantly associated with reduced recovery time, while the other groups showed no significant reduction or even increased recovery time.

**Table 4:** Recovery time length association with early/late/no drug use

	Drug use	Beta (95% CI)	*P*-value
Arbidol	Early use vs No use	− 5.2 (− 7.5, − 3.0)	4.0E−06
	Late use vs No use	1.4 (− 0.1, 2.9)	0.074
	Overall use vs No use	− 0.1 (− 1.8, 1.6)	0.91
Ribavirin	Early use vs No use	− 0.3 (− 2.2, 1.59)	0.75
	Late use vs No use	3.0 (1.1, 4.8)	0.0018
	Overall use vs No use	2.1 (0.5, 3.7)	0.01
IFN-α	Early use vs No use	− 0.2 (− 2.0, 1.6)	0.82
	Late use vs No use	2.2 (− 0.1, 4.4)	0.06
	Overall use vs No use	1.4 (− 0.6, 3.4)	0.17
Lopinavir–ritonavir	Early use vs No use	4.7 (3.1, 6.2)	1.8E−08
	Late use vs No use	7.5 (5.4, 9.6)	1.7E−11
	Overall use vs No use	4.1 (2.3, 5.9)	1.4E−05

Association test between patients’ recovery time length against where early drug use or not; late drug use or not. Multivariate linear regression model was used and risk factors and confounding factors were adjusted

The findings were similar to the in-hospital time (Additional file 1: Table S2). No serious adverse events were reported. A small number of patients taking lopinavir–ritonavir reported gastrointestinal adverse effects, such as nausea, vomiting and diarrhea.

### Early use of drugs

We noticed that there was a difference in the treatment start date between patients. To evaluate whether early use is better than late use, we tested the association between the outcome and the time of starting treatment since symptoms onset in drug-specific sub-cohort. We tested the association for the primary outcome using a logistic regression model, and we tested the association for recovery time using a linear regression model.

The results are shown in Additional file 1: Table S3. There were no significant associations between the primary outcome and the drug-use start date, which may indicate no evidence for referring early use. Please also note that the sample sizes for arbidol and ribavirin were small.

However, there were significant associations between recovery time and the days when patients started using the four drugs. This suggests that early intervention is better than late intervention. Similar result was found for in-hospital time.

## Discussion

At the early occurrence of SARS-COV-2, Shenzhen had the first confirmed cases in January, 2020. Since then, 462 cases were confirmed till May, 2020 with the majority detected in February. During that period, we had little information about the SARS-CoV-2 virus and how to treat infected patients was completely unknown. Drug repurposing was considered one option and patients were given antiviral drug intervention in our clinical study.

In this retrospective study, 403 adult patients with moderate symptoms were included as the main cohort. 30 mild cases were not included, as none of them developed severe illness before recovery, making it difficult to examine the efficiency of antiviral drugs. Antiviral drugs were common interventions, including arbidol, interferon alpha-1b, lopinavir–ritonavir and ribavirin. Despite this, 90 patients developed severe disease progression and 16 progressed to critical illness requiring mechanical ventilation and ICU admission. Three mortality cases were reported, two in the middle of February and one in the late February. We evaluated the drug treatment efficiency by testing the association between the risk of developing severity and drug treatment. The antiviral drugs arbidol and interferon alpha-1b showed promising results in reducing the severity rate, using both adjusted and unadjusted regression models.

With a median hospital stay of 21 days (IQR: 15–28), 400 of the 403 patients were discharged from hospital; the in-hospital stay was longer for severe patients, with a median of 32.5 days (IQR: 22–29.5), than for non-severe patients, with a median of 19.5 days (IQR: 15–26). We tested whether drug intervention accelerated patient recovery. Overall, no drugs apparently reduced the time of patient recovery and hospital stay; moreover, lopinavir–ritonavir and ribavirin increased the recovery time. The reason is still unclear.

There was variation in the start time of taking drugs. Hung et al. claimed the potential efficacy of early intervention [17]. We separated the treatment group into an early-use group (taking drugs within 7 days of symptoms onset) and a late-use group (taking drugs after 7 days of symptoms onset); and compared their recovery time against control group. The analysis showed that the early use of arbidol may reduce the patients’ recovery time by 5.2 days (95% CI: 3.0 to 7.5) and hospital stay by 4.3 days (95% CI: 2.1 to 6.4). The late use of arbidol did not show a significant difference, although the trend was still positive. Early or late use of interferon alpha-1b did not show a significant difference. Early use of ribavirin did not show a significant difference, while late use increased the recovery time. Both early use and late use of lopinavir–ritonavir increased recovery time.

There are several limitations to this study. This cohort had three mortality cases and is thus limited in terms of evaluating death risk. The start and end time of taking drugs were different, which may introduce bias to the estimation. Drug-drug interactions may exist, and their nonlinear effect may bring bias. The estimation of drug efficiency may be further biased by interaction between unmeasured confounding factors and the administration of the drugs which is the limitation of the retrospective cohort study, but since little information was known about the disease, this observational study is still in good confidence of evidence.

## Conclusions

This retrospective cohort study demonstrated that arbidol and interferon alpha-1b contribute to reducing the risk of developing severity in moderate COVID-19 patients. The early use of arbidol within 7 days of symptoms onset may reduce patients’ recovery time.

## Supplementary Information

Additional file 1: Table S1. Secondary outcome (time to ICU) association with drug uses. Survival analysis was performed to test the association between the secondary outcome and drug use by the Cox proportional hazard model. **Table S2**. In-hospital time length association with early/late/no drug use. Association test between patients’ hospital stay length against where early drug use or not; late drug use or not. **Table S3**. Association between outcomes and the start time (in days since symptom onset) of taking drugs. Primary outcome and recovery time were compared with drug uses. **Table S4**. Sensitivity analysis for drug-drug interaction evaluation in multivariate regression analysis with primary outcome. The main effects are also adjusted. **Table S5**. Stratified analysis of comparing single drug group vs no drug group. The numbers of patients were summarized for each group and association test with primary outcome was performed.

## Data Availability

The datasets used during the current study are available from the corresponding author on reasonable request.

## Abbreviations

COVID-19: Coronavirus disease 2019
SARS-CoV-2: Severe acute respiratory syndrome coronavirus-2
IFN-α: Interferon alpha-1b
LPV/r: Lopinavir and ritonavir
CI: Confidence interval
IQR: Interquartile range
ICU: Intensive care unit
