# Supplementary material for: Antiviral drugs arbidol and interferon alpha-1b contribute to reducing the severity of COVID-19 patients: a retrospective cohort study
Source: Virol J. 2021 Jul 8;18:142. doi: 10.1186/s12985-021-01617-w (PMC8264997; doi:10.1186/s12985-021-01617-w)
Supplement: Supplementary file 1 — Secondary outcome (time to ICU) association with drug uses. Survival analysis was performed to test the association between the secondary outcome and drug use by the Cox proportional hazard model. Table S2. In-hospital time length association with early/late/no drug use. Association test between patients’ hospital stay length against where early drug use or not; late drug use or not. Table S3. Association between outcomes and the start time (in days since symptom onset) of taking drugs. Primary outcome and recovery time were compared with drug uses. Table S4. Sensitivity analysis for drug-drug interaction evaluation in multivariate regression analysis with primary outcome. The main effects are also adjusted. Table S5. Stratified analysis of comparing single drug group vs no drug group. The numbers of patients were summarized for each group and association test with primary outcome was performed. [file 12985_2021_1617_MOESM1_ESM.docx]

Additional file 1: Table S1: Secondary outcome (time to ICU) association with drug uses.

|  | Hazard Ratio (95% CI) | P-value |
| --- | --- | --- |
| Age (years) | 1·07 (1·003, 1·15) | 0·039 |
| Sex (Female) | 0·66 (0·14, 3·08) | 0·60 |
| Respiratory symptom on set | 15·7 (3·60, 67·4) | 0·0002 |
| Arbidol | 0·39 (0·02, 8·23) | 0·54 |
| Ribavirin | 0·11 (0·007, 1·70) | 0·11 |
| IFN-α | 1·64 (0·29, 9·16) | 0·57 |
| Lopinavir-ritonavir | 0·26 (0·05, 1·38) | 0·11 |

Additional file 1: Table S2: In-hospital time length association with early/late/no drug use.

|  | Drug use | Beta (95% CI) | P-value |
| --- | --- | --- | --- |
| Arbidol | Early use vs No use | -4·3 (-6·4, -2·1) | 1·3E-04 |
|  | Late use vs No use | 0·5 (-1·0, 2·0) | 0·48 |
|  | Overall use vs No use | -0·8 (-2·3, 0·7) | 0·30 |
| Ribavirin | Early use vs No use | 0·0 (-1·9, 1·9) | 0·99 |
|  | Late use vs No use | 2·9 (1·1, 4·7) | 0·002 |
|  | Overall use vs No use | 2·0 (0·6, 3·5) | 0·007 |
| IFN-α | Early use vs No use | 1·4 (-0·4, 3·2) | 0·13 |
|  | Late use vs No use | -0·1 (-2·3, 2·2) | 0·97 |
|  | Overall use vs No use | 1·7 (-0·1, 3·5) | 0·054 |
| Lopinavir-ritonavir | Early use vs No use | 4·8 (3·2, 6·4) | 4·0E-09 |
|  | Late use vs No use | 4·4 (2·3, 6·5) | 4·6E-05 |
|  | Overall use vs No use | 3·6 (2·0, 5·3) | 1·5E-05 |

Additional file 1: Table S3: Association between outcomes and the start time (in days since symptom onset) of taking drugs.

|  | Primary Outcome | | | | Recovery time | | | |
| --- | --- | --- | --- | --- | --- | --- | --- | --- |
|  | Odds ratio (95%CI) | p | Adj odds ratio (95%CI) | p | Beta (95%CI) | p | Adj Beta (95%CI) | p |
| Arbidol | 0·87 (0·71,1·09) | 0·23 | 0·90  (0·70, 1·16) | 0·42 | 0·60  (0·47, 0·74) | 4·6E-15 | 0·48  (0·32, 0·63) | 1·6E-08 |
| Ribavirin | 0·95  (0·86, 1·04) | 0·23 | 1·06  (0·88, 1·28) | 0·52 | 0·77  (0·55, 0·99) | 6·5E-10 | 0·39  (0·24, 0·54) | 1·4E-06 |
| IFN-α | 1·004  (0·95, 1·07) | 0·89 | 0·98  (0·87, 1·10) | 0·71 | 0·73  (0·49, 0·98) | 1·0E-08 | 0·64  (0·48, 0·79) | 1·0E-14 |
| Lopinavir-ritonavir | 0·99  (0·91, 1·08) | 0·79 | 0·96  (0·84, 1·09) | 0·54 | 0·66  (0·40, 0·91) | 6·2E-07 | 0·75  (0·58, 0·89) | 2·0E-16 |

Additional file 1: S4: Sensitivity analysis for drug-drug interaction evaluation in multivariate regression analysis with primary outcome.

| Drug * Drug interaction | P-value |
| --- | --- |
| Arbidol * Ribavirin | 0.99 |
| Arbidol * LPV/r | 0.99 |
| Arbidol * IFN-α | 0.83 |
| Ribavirin * LPV/r | 0.44 |
| Ribavirin * IFN-α | 0.99 |
| LPV/r * IFN-α | 0.27 |

Additional file 1: Table S5: Stratified analysis of comparing single drug group vs no drug group.

|  | No drug | Only Arbidol | Only Ribavirin | Only LPV/r | Only IFN-α |
| --- | --- | --- | --- | --- | --- |
| No. of Patients | 51 | 7 | 1 | 4 | 54 |
| No. of severity | 42 | 0 | 0 | 0 | 15 |
| No. of Non-severity | 9 | 7 | 1 | 4 | 39 |
| P-value* | - | 3.8 E-05 | 0.19 | 0.0020 | 1.9 E-08 |

* Fisher exact test for each single drug group vs No drug group

|  | Hazard Ratio (95% CI) | p-value |
| --- | --- | --- |
| IFN-α Only vs control* | 0.25 (0.11, 0.41) | 0.006 |

*“Only IFN-α” group (54 patients) vs “No drug” group (51 patients) using the multivariate regression (adjusting age, sex, fever, fatigue, cough and disease history).
